# Mapping (very) social epistemology

**DOI:** 10.1007/s44204-026-00444-6

**Published:** 2026-08-08

**Authors:** Neil Levy

**Affiliations:** 1 Department of Philosophy, Macquarie University, Sydney, NSW 2109 Australia; 2 Uehiro Oxford Institute, University of Oxford, Oxford, OX1 1PT UK

**Keywords:** Social epistemology, Group knowledge, Non-ideal epistemology, Philosophy of science

## Abstract

A map of a debate or conceptual domain is a representation that allows us to see the relationship between some of its elements. A good map can increase our understanding of the debate. In this paper, I offer a very partial map of some elements of social epistemology. I argue that social epistemology can be divided into more kinds than previously recognized and that these kinds are interestingly different from one another. I use the distinctions drawn to make further distinctions, arguing that some instances of social epistemology that seem to be examples of non-ideal social epistemology should instead be seen as examples of ideal theory.

A good map represents a territory in a way that helps us to navigate it better. A map of an area of inquiry can be judged against the same sort of standard. To navigate a conceptual landscape is to be able to find one’s way around in it, to be able to orient oneself. A good conceptual map increases our understanding.

In this paper, I’m going to offer a very partial map of social epistemology, with the aim thereby of allowing us to navigate it a little better.^1^ There are many, many ways we could map the terrain. I won’t even try to mention all of them. Some of those that go unmentioned might be illuminating. My ambition is merely to highlight one that I take to be underappreciated and thereby further our understanding of the now familiar distinction between ideal and non-ideal social epistemology. I begin with that distinction, before going on to introduce and defend the utility of a different distinction, between different kinds of what Alexander Bird (Bird, 2010, 2023) has called social-social epistemology. I will argue that there are important differences between these kinds of social-social epistemology. Social epistemology may socialize either the agent or their cognitive processes (or, of course, both). I will also show that both kinds of social-social epistemology come in ideal, as well as non-ideal, forms, and that a theory that makes explicit empirical commitments is not always a non-ideal theory.

## Ideal and non-ideal social epistemology

Ideal theory abstracts away from the messy realities of the world in order to describe what it takes to be essential features of its subject matter. Until recently, most epistemology has been ideal theory: it has largely ignored differences between agents and differences in how agents are socially or physically situated in the world. It has also ignored the realities of cognitive processes, such as the biases and psychological quirks of animals like us. Non-ideal epistemology aims to capture the epistemic differences that differences between agents or the realities of cognitive processes make to knowledge acquisition and transmission. Motivated largely by the concern that the pursuit of idealized norms may be counterproductive, or that idealized models of epistemic agency obfuscate epistemic injustices, it aims to develop an epistemology that is attentive to social setting or status, or to the ways in which the cognitive processes that underwrite knowledge acquisition and transmission actually work.

The ideal/non-ideal theory distinction is not the distinction between theories that idealize and those that do not. Some degree of idealization is necessary for theorizing at all (McKenna, 2023). Theories are intended to aid us in understanding the world, and they need to abstract from its complexity in some respects or to some degree to achieve this goal. Equally (and as we’ll see), some degree of realism is not sufficient to make a theory non-ideal. The ideal/non-ideal distinction marks points along a continuum, not discrete categories. As McKenna also stresses, there’s nothing wrong, in principle, with ideal theory. Ideal theory obfuscates when it is inappropriately idealized, given its goals. Ideal epistemology may be entirely appropriate for, say, analyzing the concept of knowledge, but the same ideal theory might become obfuscatory when it is taken to provide practical guidance for members of minoritized groups.

Ideal epistemologists aim (often implicitly) to describe and illuminate how *agents in general* acquire, retain, and transmit knowledge. Non-ideal theorists are concerned with the differences that differences make. Non-ideal theories are thus intended to apply more narrowly: to specific groups of people in specific circumstances.^2^ This narrowness of applicability may be demographic; that is, the concern may be with distinct challenges faced by (or advantages of) specific social groups. Alternatively, it may be modal, intended to capture how people at this world differ from rational agents in other possible worlds, with different psychological dispositions to those distinctive of humans. This difference in how broadly applicable a theory is intended to be yields a test for assessing the degree to which a theory counts as ideal: if it is intended to apply very broadly (say, to the rational inhabitants of this world and any nearby world in which there is rational agency), it is an ideal theory. The narrower the range of agents it is intended to apply to, the stronger its claim to being non-ideal.

This way of drawing the distinction has an upshot that is somewhat surprising: the distinction between ideal and non-ideal theories cross-cuts the distinction between theories constructed from the armchair and those motivated by empirical findings. I think that’s the right result: non-ideal theories pay attention to how the world is taken to be, and people sometimes construct theories about how the world is from the armchair. For example, an epistemological theory that turned on what used to be called ‘women’s intuition’ would be a non-ideal theory (though not, I think, a very good theory). Of course, one may draw distinctions as one likes, but I think this way of dividing up the territory will prove fruitful.

McKenna (2023) points out that the distinction between social and traditional epistemology is orthogonal to the distinction between non-ideal and ideal epistemology. Social epistemology is concerned with interactions between individuals and with groups. Nothing in that concern requires it to work with rich or empirically informed models of individuals, of groups, or of their interactions. The move from debates over the conditions under which *S* knows that *p* to debates over when *S* knows that *p* due to testimony from S* is hardly a move toward non-ideal models.

Thus, the social/traditional epistemology distinction cross-cuts the non-ideal/ideal distinction. We might generate a 2 × 2 matrix to represent these distinctions, which I’ve populated with examples (a relatively random selection of the many important contributions to epistemology we might situate in the matrix).

**Table:** 

	Social	Individual
Non-ideal	(Graham, 2024; Shieber, 2012)	(Bishop & Trout, 2004)
Ideal	(Coady, 1992; Fricker, 2006)	(Gettier, 1963; Goldman, 1976)

There are various ways we might hope to enrich this very sketchy map. We might use a scatter plot rather than a matrix in order to represent where on the ideal/non-ideal continuum a view is best situated. We might add more dimensions to our matrix. We might, for example, add a dimension to the matrix representing the formal/informal distinction. Formal epistemology, like informal, can be more or less idealized. Whether adding this, or some other, dimension would contribute significantly to our understanding of the conceptual landscape remains to be seen. It’s unlikely to prove as fecund as was drawing attention to the ways in which the ideal/non-ideal distinction cross-cuts the social/individual distinction, because doing so illuminated the risk that social epistemology runs of obscuring social facts and of yielding prescriptions that are not merely unsuitable for the actual circumstances we find ourselves in, but would actually exacerbate epistemic problems. Ideal theory too easily functions ideologically (Mills, 2005).^3^

There is, however, a case for highlighting a different distinction between kinds of social epistemology.

## Two varieties of social-social epistemology

Alexander Bird (Bird, 2010, 2023) has argued that the ‘social’ dimension of social epistemology can come in two flavours. Social epistemology can be concerned on the one hand with the social environment in which individual agents are embedded, and on the other hand, it can socialize the agent itself.^4^ The first, exemplified by traditional work on testimony but also by most virtue epistemological social epistemology, Bird calls individual-social epistemology. The second, exemplified by work on group epistemic agency (e.g. Lackey, 2021; Tollefsen, 2002, and of course Bird himself), he calls social-social epistemology.^5^

The distinction Bird points to is a genuine one, but it risks obscuring as much as it illuminates. There are approaches to knowledge and belief that deserve to be called social-social epistemology, though they are *not* concerned with groups as epistemic agents. This work cannot be perspicuously represented using Bird’s distinction as it stands.

The social-social epistemology I have in mind does not socialize the agent, in the sense of focusing on a group agent to which individuals belong and partially constitute. Rather, it socializes the agent by arguing that the cognition of individual agents is importantly social. Bird’s social-social epistemology might therefore best be called social^(group)^-social epistemology, whereas the kind I have in mind might be called social^(cognition)^-social epistemology.^6^

As a way in to understanding what social^(cognition)^-social epistemology is, and what it isn’t, let’s begin with recent work that is explicitly presented as a contribution to individual-social epistemology, Molly O’Rourke-Friel’s recent paper about the conditions under which *S* knows that *p*. On O’Rourke-Friel’s view, *justification* is constitutively social. She points to Mercier and Sperber’s (Mercier & Sperber, 2011, 2017) empirical and conceptual case for the claim that human reasoning is distributed across multiple agents. Mercier and Sperber argue that individuals are each biased in favour of their own opinions, but in a social context (with sufficient diversity), these biases conduce to the exploration of conceptual space and the identification of the strongest arguments. In the light of this evidence, O’Rourke-Friel argues that individual justification is dependent on interaction; often *S* knows that *p* only because *S* has exchanged reasons with S*, and without these exchanges, *S* would be unable to know. On this basis, she thinks of social epistemology not as a sub-field of epistemology concerned with different questions but rather as ‘a methodological commitment that should reorient our attitudes towards the questions posed, and the answers offered by, traditional, individualistic epistemology’ (O’Rourke-Friel, 2025: 20).

Because O’Rourke-Friel is (for the purposes of her paper at least) agnostic on the existence of group attitudes, she sees her account as a contribution to individual-social epistemology. She is, of course, right that her account is not an instance of Bird’s social-social epistemology. It is not social^(group)^-social epistemology. It might, however, count as social^(cognition)^-social epistemology. We see this most clearly if we depart somewhat from her account, strengthening the extent to which cognition is socialized. That yields a clear variant of social^(cognition)^-social epistemology, perhaps a strong variant (compared to which we might see her account as weak social^(cognition)^-social epistemology).

On O’Rourke-Friel’s account, agents owe the justification of their individual beliefs to group deliberation. Justification depends on the exchange of reasons, but it is *individuals*’ beliefs that change (that’s why she categorizes her account as individual-social epistemology). The interaction is essential only because the individuals are unable to generate these reasons, or to put them to the kind of test required to make their beliefs knowledgeable, on their own. Mercier’s experimental work provides evidence that group deliberation *can* work like this: after the exchange of reasons, individuals change their minds in response to the strongest arguments (Mercier et al., 2017; Trouche et al., 2015). But the deliberative exchange need not conform to this picture to produce knowledge.

O’Rourke-Friel describes this exchange as deliberative. As she would no doubt acknowledge, however, it is often highly conflictual. Unsurprisingly, given it is often conflictual, the exchange of reasons does not always end with participants converging in their views. Far from it. The paradigm Mercier uses might give rise to the impression that deliberation leads to agreement, but (for very good reasons) Mercier used the Wason selection task in his studies, a task on which it is quite easy to demonstrate why the right answer is correct.^7^ Deliberation about questions with clearly determinate and demonstrably correct answers is relatively rare in public life, of course. We do not often argue about the correct solution to the Wason selection task, or items on the cognitive reflective test, or problems in arithmetic. Rather, we argue about policy, or values. What should our immigration policy be? How much should we spend on welfare? On foreign aid? Should that new housing estate go ahead? How should we respond to climate change? Notoriously and obviously, people do not always converge on the answers to these questions after the free exchange of reasons. Though people do seem to respond to good arguments (Coppock, 2023), they often move far too little for the result to be convergence.

Perhaps there are good Bayesian reasons for such a lack of convergence: different priors can give rise to rational polarization on the same body of evidence (Dorst, 2023; Westfall, 2024). Strong arguments and good evidence may not even propositionally justify convergence. For all that, the exchange of reasons, including the conflictual exchange of arguments, may be epistemically fruitful. It may produce epistemic benefits *without* changing minds.

Science, our most successful epistemic practice, is a social enterprise, with scientific research distributed across multiple individuals and laboratories. As philosophers of science (including Bird (2023) himself) have stressed, however, science produces knowledge without always changing the beliefs of many individual scientists. The strongest argument does not always change minds: rationally or irrationally, scientists may fail to be convinced. Think of the controversy over tectonic plate theory: while it may be a myth that the scientific community closed ranks against Wegener’s continental drift theory (Vickers, 2023), there can’t be any doubt that some scientists were slow to accept the theory despite the evidence.

Perhaps there are even cases in which the scientific community as a whole has closed ranks against a theory in the face of sufficient evidence; actual cases are difficult to interpret (in some, for example, the bacterial causes of stomach ulcers, the response was mixed and arguably appropriate (Atwood, 2004); in others, for example Lysenkoism, it’s difficult to separate out genuine conviction from political expediency). Whether or not such cases exist, there are certainly instances where science produces new knowledge while changing few minds. Even if scientific groups are not epistemic agents in their own right, the study of the epistemic properties of such scientific knowledge counts as social^(cognition)^-social epistemology, of a stronger sort than O’Rourke-Friel has in mind because the relevant knowledge is possessed despite few individuals knowing it.

These are cases within the domain of social^(cognition)^-social epistemology because they involve knowledge generation in which the processes are distributed across multiple individuals; insofar as few changes of minds are involved, moreover, they are instances of *strong* social^(cognition)^-social epistemology. There are cases of scientific knowledge which represent even stronger social^(cognition)^-social epistemology; in these instances, knowledge is produced that *no* individual knows.

To home in on this sort of scientific knowledge, it’s worth taking an excursion through Bird’s case for social-social epistemology because he, too, is after a sort of knowledge that need not be known to any individual. His case for this sort of knowledge fails, I believe (or at best yields a kind of knowledge that is epistemically insignificant). But its failure allows us to identify the properties that knowledge must have if it is simultaneously unknown to any individual and yet epistemically significant.

Bird argues that a scientific discovery can be known by a collective such as a nation or even what he calls ‘wider science’ even when no individual is so much as aware of its existence. Suppose (to use his own example) Dr N publishes a paper in a journal reporting a finding or result that finds few readers and no immediate uptake. Dr N then dies, as do the reviewers for the journal, the handling editor, and everyone else who has read the paper. Some years later, Professor O comes across the paper and cites it in her own influential research. As a result, Dr N’s discovery comes to be widely known. Bird argues that Dr N’s finding is known to wider science at every moment after its publication, not just at times at which there were living people who were aware of it. As long as the knowledge is in the public domain (not locked up in a file drawer or a safe, say), it is part of scientific knowledge.

Perhaps Bird is right that there’s some sense in which findings that are merely accessible count as part of scientific knowledge. But any sense in which that’s true is very weak. Dr N’s discovery plays no actual role in science or in the life of the broader communities to which scientists belong. It does not guide inferences or make a difference to institutions and practices. The discovery is neither occurrent knowledge, nor the kind of dispositional knowledge that plays a background role in actual inference, nor the kind of dispositional knowledge that given the right triggers would come to be immediately occurrent, or immediately come to play a background role in inference. Given these facts, there are grounds for thinking that any sense in which Dr N’s discovery is known by science is epistemically insignificant.

To drive the point home, we might compare Dr N’s discovery with other true scientific claims that appear *not* to be known by science. Right now, there are innumerable true claims that can be inferred from existing findings, where these findings *are* known by science if anything is. All it would take is for someone to put these findings together. That’s no trivial task, of course: it’s not putting 2 + 2 together. But it’s the kind of task that researchers accomplish all the time. Most of these true claims are not important, but some of them are very important. Are these important claims known by science? They appear not to be. It is probably true, for example, that right now some people are sicker than they would be were some of these inferences to be made. It would be very odd indeed to say that doctors are failing to act on claims known to science in not treating these patients. On what grounds should we count Dr N’s discovery as known by science when these claims are not?

Bird (2023: 90) notes that ‘accessibility comes in degrees’. During the Middle Ages, he points out the knowledge of the ancient Greeks was accessible only with difficulty by European scholars, since exchanges between them and the Islamic scholars who preserved this knowledge were infrequent. Bird describes this as a borderline case. Insofar as a claim is accessible to a scientific community, it is known by that community; Dr N’s discovery is highly accessible because it is published in an easily available journal that is indexed by the major services. As soon as Professor O searched for information like that for her own research, she found his paper. So Bird might say that something counts as known by science at *t* if (inter alia) it would quickly be accessed by someone with the right skills and motivations. But these conditions are surely satisfied by many of the scientific claims mentioned above, which are not known by science. Were someone with the right skills and motivations to set themselves to thinking about the question, they would quickly infer them. So easy accessibility doesn’t suffice for a claim to be known by science.

Perhaps Bird might point to differences between these cases that could entail that Dr N’s discovery is known whereas these easily accessible claims are not. Perhaps he might instead bite the bullet. In the end, the question whether Dr N’s discovery counts as known by science in the entire period from its publication to its rediscovery might be merely verbal. What matters for our purposes is that whether or not it is known, this knowledge is epistemically insignificant until it is poised to play a role in inference. A convincing case for genuine knowledge that is not known by individuals must show that that knowledge plays an actual role in cognition.

There are in fact many examples of such epistemically significant knowledge, some of which are provided by Bird himself. Bird (along with a number of other philosophers of science) has emphasized how scientific research is distributed across multiple individuals, labs, and—importantly—non-human scaffolds. As he notes, scientists’ access to and interaction with data are often entirely mediated by computers and their software (Bird, 2023: 7–10). In these cases, the computer represents information that no human may ever represent, not even dispositionally: that information does not play a direct role in the human’s own inferences and (given that the computer may be running machine learning algorithms that are entirely opaque to the scientists) there are no circumstances under which that information could be accessed.^8^ But it is epistemically significant: it plays a role in knowledge production.

Heavy reliance on computers is a relatively recent phenomenon, of course, but in many ways, this sort of reliance is a natural outgrowth of long existing scientific practices. Science has been a collective enterprise from the start. From the beginning, scientists have relied on one another to produce the tools and the findings that they then go on to use. As Bird notes, while palaeontologists understand the principles of radiometric dating, they typically take the theoretical background and its justification on trust from physicists (Bird, 2023: 85). This trust relationship between palaeontologists and physicists is typical in science: even within disciplines—even with single labs—individuals do not and often cannot reproduce the findings of other scientists; they nevertheless depend on each other. The outsourcing of representations to computers and to models is a natural extension of outsourcing to individuals; there is no difference, in principle, between depending on a representation that one cannot oneself retrieve or fully understand that is stored in the head of another scientist and depending on a representation one cannot retrieve or fully understand that is stored in a computer, or a model. In typical cases of each kind, the scientist relies on claims that they cannot themselves justify, and even on some of whose content they are unaware.

In fact, there’s a case for thinking that dependence on, and therefore the epistemic significance of, propositions that no human represents predates dependence on computers by millennia. Researchers in cultural evolution have emphasized that some processes are adaptive despite the fact that those who employ them lack true explicit beliefs about what they’re for or how they work. For example, those who endorse and abide by adaptive food taboos in Fiji have false beliefs about their justification (Henrich & Henrich, 2010), while in other cultures, people may have no belief at all about the justification of food preparation techniques (Henrich, 2015). Moreover, food preparation techniques might be distributed across multiple members of a group, with no one individual knowing the full range of procedures necessary. In some cases, they may not even know that others play an essential role in processing the food. In all these cases, the procedure itself may be said to embody knowledge—propositions about how food should be prepared—that no person represents (Levy & Alfano, 2019).

In a similar fashion, scientific procedures may embody knowledge that no agent represents. Nick Shea has noted that experimental procedures may be followed religiously, without anyone knowing why (or even whether) every step is necessary. The use of *these* chemicals, in *those* quantities, the employment of a centrifuge for *this* length of time and at *that* speed, and so on; all of these steps may have been developed by previous researchers through trial and error and inherited by lab members who continue to use them because the procedure works. The techniques and steps may be copied ‘without any appreciation of whether or why they are necessary to achieve the goal – following an experimental protocol can feel rather like following a magic spell’ (Shea, 2009: 2434). Indeed, some of the steps may be black boxes to those who employ them (what actually happens when I place the compound in the centrifuge, when I add the reagent?).

In such cases, the knowledge may have been lost to individuals or never been possessed by any individual at all. The procedure itself embodies the knowledge. These are cases of strong social^(cognition)^-social epistemology, strong because cognition is socialized in ways that escape individual understanding.

## Putting the distinction to work

Bird’s account of group knowledge is supposed to yield a social^(group)^-social epistemology because it socializes knowledge production and the agent; the group itself is said to know. While I have doubts about Bird’s account of group knowledge, there are other accounts I find more plausible (see Lackey, 2021 for an alternative account of groups as knowers). We should recognize at least the possibility of a social^(group)^-social epistemology. Our matrix should make room for both kinds.

We might add the distinctions between different kinds of social epistemology to the left-hand side of our existing matrix. Doing so would risk needless complication, however, and therefore fail to increase our understanding of the conceptual terrain. It might be more illuminating instead to provide a different representation of the debate, one that allows us to see commonalities and differences that might otherwise have been obscured. This is especially the case when we map the kinds of social-social epistemology against the ideal/non-ideal distinction. Doing so forces us to think more deeply about what it takes for a theory to count as non-ideal, with some surprising upshots.

Here’s the matrix, populated with some examples.

**Table:** 

	Social^(group)^-social	Social^(cognition)^-social
Non-ideal	(Hutchins, 1995)	(Paolo et al., 2017)
Ideal	(Gilbert, 1992; Lackey, 2021)	(Levy, 2021; O’Rourke-Friel, 2025)

It is always, of course, possible to dispute whether particular examples are classified appropriately. However, many people will find this classification not merely inaccurate but wrongheaded, on the grounds that it classifies views that are almost paradigmatically non-ideal—because they rely on empirical research—as ideal. I think that would be a mistake. A theory can be motivated by empirical research, even make empirical claims, and yet count as ideal, because it is not concerned with the difference that differences (between groups of agents or between agents in this world and those in nearby possible worlds) make.

The ideal/non-ideal distinction is not a distinction between theories that do not and those that do make empirical assumptions, nor is it the distinction between theories that idealize in certain respects and those that do not. All theories make empirical assumptions, and all idealize in certain respects. *S* knows that *p* epistemology makes a range of empirical assumptions: for example, that agents typically become aware of evidence through sense perception, and that cognition largely takes place in the head. On the other hand, non-ideal theories necessarily idealize in many respects, the better to focus on the aspects of the world the theorist is interested in understanding. For example, a great deal of work on epistemic injustice focuses on the difference that gender or race makes to the credibility of testimony, without attending to the details of cognitive mechanisms.

In the first section of this paper, I suggested that we can best distinguish ideal from non-ideal theories by reference to the set of agents, actual or possible, the theory is intended to be true of. An ideal theory is supposed to be very broadly true; the claims it makes are intended to hold for all rational agents, or (somewhat more narrowly) all agents that are recognizably like us at our best. A non-ideal theory is intended to identify processes or mechanisms that are true more narrowly: true of a particular minoritized group, for example, or (rather more broadly) of agents that share our particular psychologies.

In this light, Paolo et al. (2017) are paradigmatically non-ideal. They make claims about agents that have particular sets of psychological mechanisms, mechanisms that might be unique to animals with our sort of evolutionary history. They do not intend to develop accounts that can be assumed to hold, even in outline, in possible worlds very different from our own, or for aliens. Such creatures could, in principle, know what we know, but know it on the basis of very different processes.

I don’t think the classification of Paolo et al. (2017) as non-ideal would be controversial. It is far more surprising that my own work (Levy 2021) and O’Rourke-Friel (2025) should be classified as ideal. Both cite and build on psychological research; to that extent, one might want to claim them for the non-ideal camp. However, there’s a case for classifying them as ideal on the grounds that the psychological research they cite doesn’t play the role of delimiting the set of agents of which their views are held to be true, not, at any rate, to any extent greater than the delimitation seen in paradigmatic ideal theories.^9^

O’Rourke-Friel argues that the processes that confer justification on agents’ beliefs extend ‘to include her interactive engagement with other deliberative participants’ (2). That’s an empirical claim, of course, and she supports it by recourse to the arguments and empirical data of Hugo Mercier and Dan Sperber. She aims thereby to develop a social epistemology ‘for individuals like us’, as the subtitle of her paper would have it. Her view, therefore, seems intended to develop an account that applies *locally*, at this world, with its limited and social inhabitants. She seems thereby to occupy the same quadrant of the matrix as Paolo et al.; hers is a view for us, not for aliens, or indeed the inhabitants of worlds in which evolution took a different turn and our counterparts were much less social animals. So far, so non-ideal.

If O’Rourke-Friel is correct, then it’s true that the justification of our claims depends on processes that are distinctive of the kind of animals we are. Aliens and solitary hominids might not rely on processes that are anything like those she points to. It does not follow, however, that her view is intended to apply locally. That depends on whether she accepts a further claim: that almost any actual or possible agent capable of knowledge about the domains regarding which *we* rely on the sort of justification she points to must be ‘like us’ in also relying upon this sort of justification. Does she believe, for example, that if our evolutionary history had been different, and we’d evolved a different set of psychological features, we still would have been capable of knowledge about these domains, or does she believe (as I do) that knowledge of this sort depends on these sorts of psychological features for almost all agents with less than godlike capacities?

O’Rourke-Friel is indeed a non-ideal theorist if she thinks that justification just *happens* to be distributed for us (and that aliens and other hominids could know what we know on the basis of very different processes). She is an ideal theorist (or at any rate closer to the ideal end of the spectrum than the non-ideal) if she thinks these sorts of processes are required by almost any agent capable of knowledge of the sorts of propositions for which we rely on social processes of justification. Had our evolutionary history taken a different turn—were we, for example, solitary hominids, perhaps sharing a more recent common ancestor with orangutans than with chimpanzees—then we would not know these propositions on the basis of more intra-individual processes. Instead, we would not be capable of knowing them at all. There would be regions of possible knowledge that are closed off to us and to any agent with less than godlike cognitive powers.

A non-social being capable of knowledge about domains like these would need the capacity to possess deep understanding about multiple different sorts of domains, and possession of this capacity requires intra-individual cognitive powers that *vastly* surpass ours. Consider the cognitive powers that would be required for a solitary agent to know *that a fossil sample is approximately 3.2 million years old*. A contemporary paleoanthropologist might establish this on the basis of radiometric dating, which uses the known rate of decay of radioactive isotopes; in doing so, they rely on deeply and broadly distributed cognitive processes and states. As Bird (2023) points out, while we should expect the paleoanthropologist to understand the principles of radiometric dating, she ‘will nonetheless have to trust her physicist and geologist colleagues regarding the theory (of radioactive decay) and the details of its application (e.g. the half-life of 40 K) as well as the various assumptions made’ (6). Sufficiently detailed knowledge of two such different fields to be able to dispense with epistemic dependence and the distribution of justification is well beyond beings like us.

In fact, we cannot hope to dispense with epistemic dependence and the distribution of justification within a *single* field. Not only do paleoanthropologists rely on physicists, but paleoanthropologists also rely on other paleoanthropologists, and physicists rely on other physicists. The very physics that paleoanthropologists rely upon is not wholly understood by any one individual; rather, there, too, justification is spread across multiple specialties. Even the authors of a single paper depend upon one another for the justification of their claims, as Hardwig (1985) pointed out 40 years ago. While science offers a spectacular example of epistemic dependence, moreover, such inter- and intra-field epistemic dependence is seen in other areas of human knowledge too, such as history (Levy, 2021). For aliens to know what we know without epistemic dependence of the sort O’Rourke-Friel points to, they would need to be truly godlike.

While O’Rourke-Friel aims to develop an epistemology for ‘individuals like us’, on plausible assumptions about how we are capable of knowing a great deal of what we know, she does not develop an epistemology that applies locally. Rather, it is true very broadly: almost any kind of agent capable of knowing facts like *those* will depend on distributed justification, in the same way we do. It’s only almost godlike beings that can know without depending on distributed justification. Of course, delimiting the class of agents to which her theory applies by excluding such beings entails her theory is not as ideal as it might be: it’s not at the extreme end of the ideal/non-ideal continuum. But actual existing ideal epistemology never is at the extreme end. O’Rourke-Friel’s account seems broad enough in scope to count as a contribution to ideal epistemology.

The case for my own account being closer to ideal, rather than non-ideal, epistemology is if anything even stronger because I (explicitly) accept these sorts of claims about the deep epistemic dependence of agents upon one another. I argue that bad beliefs—beliefs that are contrary to a strong scientific consensus—are typically rationally held by agents. They are justified by the total body of evidence available to agents, especially testimony from others, where that evidence is weighted by its prior plausibility to the agent and the assessed reliability of informants. Like O’Rourke-Friel, I motivate my account by close attention to empirical research. I argue that my account is supported by work in cultural evolution (e.g. Henrich, 2015), in empirical psychology (Sher & McKenzie, 2006), and in cognitive science (Sperber et al., 2010). But the empirical claims I cite commit me to an account that has very broad scope. If these claims are true, then almost any kind of agent capable of scientific knowledge (and a great deal of extra-scientific knowledge besides) is dependent on distributed cognitive labour. This labour enables knowledge in cognitive domains that would be forever closed to any beings that are not godlike in their capacities.

Just as the social/non-social distinction dissociates (in both directions) from the ideal/non-ideal distinction in epistemology (as McKenna showed), so it is a mistake to map the non-ideal/ideal distinction onto the distinction between approaches that pay attention to human psychology and those that do not. Some of the work that McKenna (and Begby (2021) before him) is committed to counting as non-ideal epistemology highlights features of *cognition*, rather than features of the cognition of particular agents who just happen to have particular quirks. The mere fact, for example, that theorizing attends, for example, to ‘constraints arising from distinctive capacity limitations of the human mind’ (Begby, 2021: 3) does not make it non-ideal, not unless these constraints are distinctive in the sense that we should not expect other actual and possible non-godlike cognizers to share them.^10^

There is, surely, a correlation between empirical richness and non-ideality. The more facts about the world and the agent we take to be relevant to our epistemological theorizing, the more likely it is that we are developing an account that applies locally: to beings that are like us and are situated as we are, rather than an account of how knowledge is generated by rational agents. But when we aim to draw out the implications of a particular psychological disposition or process, we may be engaged in ideal epistemology: when that disposition or process is one that we ought to expect to be possessed very widely, or possessed by almost any agent capable of knowledge about a domain. This, surely, is the right result: it shows the utility of the ideal/non-ideal distinction. We already had terminology, after all, to capture the distinction between empirically informed and uninformed philosophy; we don’t need to introduce a distinction that does no new work.

In asking whether a theory is ideal or non-ideal, we should ask how broadly it applies. Using Lewisian terminology, we might ask how distant are worlds in which agents come to possess knowledge of the same sort via a different kind of route. If such worlds are very distant, and the beings in them utterly unlike us, then our work may count as ideal epistemology.

Whether or not I’m right in how I classify O’Rourke-Friel or myself—or anyone else—is not especially important for my purposes here. Rather, my aim is to demonstrate the utility of the conceptual map offered. Dividing up the terrain in the way suggested brings into focus the kind of questions I’ve highlighted. Just as McKenna’s highlighting the distinction between ideal and non-ideal social epistemology made it apparent that concern with interactions between agents isn’t by itself sufficient for a theory to count as non-ideal, so highlighting the distinction between social^(group)^-social-epistemology and social^(cognition)^-social epistemology helps us see what it takes for the latter to count as non-ideal. We might have thought that a theory is non-ideal because it pays attention to how the world is. But that may not be enough: the empirical commitments must commit the theorist to holding that the theory applies locally, either to groups within this world, or to agents relevantly like us.

Social epistemological theories may be social in different ways. They may situate the agent in a group. They may socialize the agent. And they may socialize the cognitive processes involved. The conceptual map also forces us to ask just *how* the theory is idealized, and what role its empirical commitments play. I take it that this constitutes progress, independently of whether my attempts to answer the questions it brings to light are correct.

## Conclusion

Attention to the actual powers and limitations of agents and to the actual world in which we live has brought large benefits to epistemology. A focus on oppression and domination, on cognitive limitations, on epistemic pollution, and on the workings of our institutions has all allowed us to ask new and important questions and to formulate plausible answers to these questions. It has given rise to different ways of doing epistemology and amply proven its value.

If I’m right, there are more different kinds of social epistemology than previously recognized, with interesting differences and commonalities between them. It’s important to recognize these differences because different tools are appropriate for different jobs. Ideal social epistemology remains the appropriate approach to take to certain questions, but it has limitations; it’s important to recognize, lest it obscure questions that we should ask, or offer solutions that might actually make epistemic problems worse. Those who develop theories and who seek to apply them therefore must be attentive not only to the world but also to our conceptual maps. Good maps help us to find our way; the map I’ve offered here might prove a useful navigational tool.

## Footnotes

1 ‘Map’ and ‘mapping’, as well as related terms like ‘terrain’ and ‘navigating’, are here used metaphorically, though in a way that should be familiar. A map, in this sense, is a way of representing a debate or a domain in a manner that highlights some of its features and the relationships between these features. Since a debate is not a physical entity, the map need not resemble the map of a landscape (though it can). It might consist of a spider plot, for example, or (as here) a matrix. ‘Navigating’ this terrain consists in finding one’s way about, in a metaphor familiar since at least Wittgenstein.

2 Why should theorists’ intentions matter to whether a theory is ideal or non-ideal? A standard criticism of ideal epistemology is that though it has pretensions to universality, its claims are true only of privileged knowers. Were it to present itself explicitly as (say) white male epistemology, it would not be vulnerable to that objection; nor, of course, would it be ideal epistemology. It is not the content of a theory, alone, that determines whether it is ideal or non-ideal, but how broadly it is intended to apply.

3 This is not, of course, a criticism of formal epistemology. A method or approach to epistemological questions can add to our understanding without it being true that producing a map of epistemology that represents that method or approach adds to our understanding.

4 Bird presents these approaches as distinct but as a reviewer for this journal points out, there are feminist virtue epistemologists who socialize both the agent and the environment in which the agent is embedded (e.g. Code, 2006; Pohlhaus, 2012).

5 It’s worth noting that Bird misses a quadrant of the 2× 2 matrix he commits to. In addition to individual-social and social-social epistemology, we should recognize the possibility—and the existence—of social-individual epistemology. If individual-social epistemology is concerned with the social setting of individual agents, social-individual epistemology is concerned with how individual agents interact with group agents. Any account of group epistemic agents that is non-summativist (like Bird’s own) is committed to dissociations between group doxastic states and individual doxastic states and therefore is in a position to ask questions characteristic of social-individual epistemology.

6 While I won’t explore it here, we should also recognize the possibility of social^(group)(cognition)^-social epistemology. This would describe epistemology concerned with groups that are epistemic agents *and* whose cognitive processes are socialized. It may well be that the investigation of the epistemic properties of certain sorts of organized scientific groups counts as social^(group)(cognition)^-social epistemology.

7 The Wason selection task is appropriate for these studies because it has an uncontroversially correct answer, but most people do not perform well on it. By using it, Mercier is able to produce unambiguous evidence that group deliberation can outperform individual reasoning.

8 The knowledge plays an indirect role in individual scientists’ inferences: the content plays a characteristic role in the chain leading to the scientist’s coming to believe that *p*. But there are no circumstances in which it plays a direct role: in which it serves as an explicit or implicit premise in their reasoning.

9 There are important differences between work like this and paradigmatic ideal theories, of course. First, this sort of work is naturalistic, inasmuch as it derives its conclusions from empirical research rather than from conceptual analysis. I’ve already suggested that we should avoid collapsing the naturalistic/non-naturalistic distinction into the non-ideal/ideal distinction, though of course they commonly go together. Second (and as a corollary of being naturalistic), work like this makes its empirical commitments explicit while the latter rarely does. But making one’s commitments explicit doesn’t transform what kind of work we’re doing from ideal to non-ideal. It does, however, help avoid one possible cost of ideal theorizing: a lack of awareness of their commitments can lead a theorist to overgeneralize their claims or to overlook ways in which they might be controversial.

10 As well as attending to these ‘endogenous’ constraints, Begby also points to those ‘arising from specific limitations on the information environments that epistemic agents are forced to operate within’ (3). It is an interesting question whether these ‘exogenous’ constraints can be expected to vary across actual and possible cognizers to an extent that would make any, or almost any, epistemology that is concerned with them an instance of non-ideal epistemology.
